# Disentangling Aging and Mood Effects on Emotional Memory

**DOI:** 10.3389/fnbeh.2022.944363

**Published:** 2022-08-26

**Authors:** Kylee Tamera, Courtney Kannampuzha, Viviane Ta, Pascal Hot, Patrick S. R. Davidson

**Affiliations:** ^1^School of Psychology, University of Ottawa, Ottawa, ON, Canada; ^2^Laboratoire de Psychologie et NeuroCognition (LPNC), Université Savoie Mont Blanc, Chambéry, France; ^3^Institut Universitaire de France, Paris, France

**Keywords:** mood induction, positivity effect, memory, aging, emotion, negativity bias, positivity bias

## Abstract

Older adults tend to be in a more positive mood than young adults, and tend to remember positive information more often than negative information, yet the link between their positive mood and their positive memory bias has not often been explored. In this study, we manipulated young and older adults’ moods prior to their completing an emotional memory task. For mood manipulation, young (*n* = 147) and older (*n* = 111) adults viewed a positive, negative, or neutral video lasting 3 min. To validate the mood induction, we collected self-reported ratings of valence and arousal (affective slider; Betella and Verschure, 2016) at baseline, after the video, and after the memory task. The memory task consisted of incidental encoding of 30 intermixed pictures (10 positive, 10 negative, 10 neutral valence), followed by free recall. The mood manipulation changed people’s self-reported valence, yet it did not influence self-reported arousal. The memory task revealed a consistent negativity bias in young adults. Older adults recalled negative and positive pictures equally well in all conditions. After viewing a negative video, they recalled positive pictures more often than neutral pictures, but did not show the same advantage for negative pictures over neutral pictures. This positive memory advantage was weaker in the positive mood condition. Therefore, mood manipulation influenced in part older adults’ emotional memory bias, showing some signs of mood incongruence which we discuss in terms of emotion regulation. This shows the importance of accounting for mood differences in studies on aging and memory. The robust age group differences support the view that the positivity effect in aging is the result of a negativity bias that fades with age.

## Introduction

Older adults tend to be in a more positive mood than young adults and tend to remember positive information more often than negative information. Yet the link between their positive mood and their positive memory bias has rarely been explored. From middle age to older adulthood, positive affect seems to increase (Kunzmann et al., 2000; Gana et al., 2015) and negative affect seems to decrease, although health and family situation may play a role (Hellevik, 2017). In parallel, older adults often remember positive information more than negative information when compared to young adults (Reed et al., 2014). This aging positivity effect in memory is often explained by Socioemotional Selectivity Theory (SST; Carstensen et al., 1999; Charles et al., 2003). According to SST, older adults prioritize emotional meaning and satisfaction because they are more present-oriented than young adults who are more future-oriented (Carstensen, 1992, 2006, 2019; Carstensen et al., 1999). The increase in present-oriented goals and adaptive emotion regulation may lead older adults to prioritize and remember positive information over negative information. In contrast, young adults often show the opposite, that is, a negative memory bias (Baumeister et al., 2001; Reed et al., 2014).

Despite a lot of evidence supporting SST (see meta-analysis Reed et al., 2014), the possible confounding effects of mood on the positivity effect have rarely been considered. Indeed, mood can influence a person’s memory and other cognitive functions (for reviews, see Blaney, 1986; Bower, 1987; Eich et al., 2008). It can be understood as a background affective tone capable of influencing how brief emotional experiences are processed and remembered (Rottenberg et al., 2007). Mood may sometimes be used interchangeably with affect and emotion, and considered synonymous (Knight et al., 2016). Information that is emotionally congruent with a person’s mood may receive greater attention and more elaborative processing than neutral or emotionally-incongruent material (Eich et al., 2008). This can lead to mood-congruent memory (MCM) effects, whereby participants who are in a positive mood at encoding better remember positive stimuli, and participants who are in a negative mood at encoding better remember negative stimuli. The MCM effect seems strongest when mood during encoding matches the affective tone of the target stimuli, however, it can also occur during other stages of memory processing, for instance during retrieval (Singer and Salovey, 1988; Ward et al., 2021).

In past studies of the aging positivity effect in memory, older adults often reported being in a more positive mood than young adults at the start of experiments. For instance, older adults have reported higher positive affect (e.g., Mather and Knight, 2005), lower negative affect (e.g., Charles et al., 2003; Grühn et al., 2007; Spaniol et al., 2008), or both (e.g., Mather and Knight, 2005; Emery and Hess, 2008, 2011; Fernandes et al., 2008; Tomaszczyk et al., 2008; Fung et al., 2010) when compared to young adults. Moreover, older adults have often reported fewer depressive symptoms than young adults (Charles et al., 2003; Mather and Knight, 2005; Mickley and Kensinger, 2009; Fung et al., 2010). So far, researchers have typically examined the influence of mood on the positivity effect by including mood as a covariate in their analysis. The results are divergent and inconclusive. Mood has sometimes correlated with memory (Charles et al., 2003; Barber et al., 2016), and mediated some of the effects of age on memory (Emery and Hess, 2008), whereas other times it did not (Mather and Knight, 2005; Fernandes et al., 2008; Tomaszczyk et al., 2008). It is difficult to determine the source of inconsistent findings across studies when the procedures have varied enormously (e.g., encoding task/instructions, memory test delay and type).

Even better than the existing correlational observations would be experimental manipulation of mood that minimizes the risk of error variance. One of the few studies to use an experimental mood manipulation found partial support for the mood-congruent hypothesis (Knight et al., 2002). In that study, a sad mood induction did not alter recall of negative words in young or older adults, however, it did lead to older adults recalling fewer positive words than when induced into a neutral mood. Yet, there was no positive mood induction condition, to determine whether a positive mood elicits a positive memory bias. A related study (Zhang et al., 2021) found some evidence of mood congruence using a semantic memory task (i.e., Deese-Roediger-McDermott paradigm). Positive mood induction led to greater true recognition of positive words in young and older adults. A full experimental design contrasting both positive and negative mood inductions and their effects on memory recall for positive, negative, and neutral stimuli is needed to test the interaction between mood and emotional memory in aging.

### Current Study

The goal of the current study was to directly test the mood-congruent hypothesis of the positivity effect. To exert greater experimental control, we used a mood induction protocol in which participants’ exposure an emotion-eliciting video could be controlled and randomized. We manipulated young and older adults’ mood using positive, negative, or neutral video clips. The effectiveness of the mood manipulation technique was assessed using self-reported valence and arousal (affective sliding scale; Betella and Verschure, 2016).^1^ We used an experimental design that would independently maximize MCM and positivity effects to increase the power of analysis. First, we tested memory using free recall because this seems more likely to encourage substantive (elaborative) processing, leading to MCM effects, than memory recognition tests. Free recall encourages an elaborate processing strategy whereby many encoding and retrieval cues can facilitate memory, mood being one such cue (Eich et al., 2008). Free recall also may lead to stronger emotional biases, including the positivity bias, than recognition tests (e.g., Charles et al., 2003; Tomaszczyk et al., 2008; Reed et al., 2014). Second, mood induction occurred immediately prior to the encoding of emotional stimuli because encoding congruence (i.e., congruence between mood at encoding and the stimuli) produces more reliable MCM effects and as reviewed above, mood at encoding may differ between young and older adults. Third, we presented the emotional and neutral pictures in a mixed order to encourage the distinctive processing of emotional items (Talmi et al., 2007; Talmi and McGarry, 2012; Ack Baraly et al., 2019).

According to the mood-congruent hypothesis, a mood-congruent memory advantage should appear for both young and older adults. Both age groups should show increased memory for positive stimuli relative to negative and neutral stimuli when in a positive mood, and increased memory for negative stimuli relative to positive and neutral stimuli when in a negative mood. According to SST, these relative emotional memory effects should vary as a function of a person’s time perspective whereby those who are more present-oriented (typically older adults) would exhibit a positivity bias and those who are more future-oriented (typically young adults) more of a negativity bias. Thus, older adults would show a positive memory bias that correlates with time perspective and emotional regulation, irrespective of mood. The current study tests predictions of the mood-congruent hypothesis and SST by gathering measures of mood, time perspective and emotion regulation.

## Materials and Methods

### Participants

The final sample comprised 147 young adults (17–27 years) and 111 older adults (56–91 years). The target number was 110 per age group to obtain 0.80 power to test the within-between interaction based on *a priori* power analysis for a small effect ($\eta_{p}^{2}$ = 0.01) using G*Power software (Faul et al., 2009).^2^ Data from one additional older adult participant were excluded because of an incomplete session. One young adult did not respond to all questionnaires, but completed the mood induction and memory tasks so data were retained. Two additional older adults were excluded for reporting a range of psychiatric and neurological conditions on a health questionnaire (i.e., hospitalization for mental problems, electroshock therapy, vision problems, drug abuse, schizophrenia). Participants were also screened for depressive symptoms using the Center for Epidemiologic Studies Depression scale (CES-D; Radloff, 1977) and cognitive impairments (older adults only) using the Montreal Cognitive Assessment (MoCA; Nasreddine et al., 2005). There were no outlying scores (as calculated in SPSS) for CES-D and MoCA so remaining data were retained. Participants were randomly assigned to one of the three video conditions (neutral video, negative video, positive video). Demographic and questionnaire data per age group and video condition are presented in Table 1.

**TABLE 1:** Questionnaire data for young and older adults by mood condition.

	Young			Older		
		
	Mood condition					
	Neutral	Negative	Positive	Neutral	Negative	Positive
*n*	55 (40F, 15M)	48 (35F, 13M)	44 (26F, 18M)	38 (27F, 11M)	39 (29F, 10M)	34 (23F, 10M, 1NB)
*Age	18.38 (1.39)	19.29 (1.69)	18.80 (1.77)	70.63 (7.35)	69.87 (6.64)	71.62 (6.67)
*Education	12.38 (0.68)	12.92 (1.40)	12.64 (1.18)	17.08 (2.50)	17.23 (3.08)	16.24 (2.97)
^†^MoCA	–	–	–	27.92 (2.01)	26.64 (2.29)	26.76 (2.26)
*CES-D	19.33 (9.93)	18.27 (10.53)	18.44 (10.55)	7.66 (6.20)	8.49 (7.10)	10.97 (9.64)
*FTP-total	52.02 (8.73)	50.63 (9.60)	51.40 (9.41)	39.66 (11.11)	40.21 (12.69)	38.21 (14.73)
*FTP-ambiguous	16.89 (5.53)	14.77 (5.82)	15.09 (4.13)	12.74 (4.08)	13.03 (4.45)	13.50 (5.00)
ERQ-appraisal	29.82 (6.75)	30.21 (6.89)	29.33 (5.07)	32.03 (6.45)	30.92 (5.53)	30.50 (5.97)
*ERQ-suppression	15.18 (4.51)	15.33 (5.73)	14.23 (4.57)	11.63 (4.69)	12.74 (4.94)	12.62 (4.77)

*F, female; M, male; NB, non-binary. Mean and SD for age (in years), education (in years), Montreal Cognitive Assessment (MoCA), Centre for Epidemiologic Studies Depression scale (CES-D), Future Time Perspective total score (FTP-total), Future Time Perspective ambiguous subscore (FTP-ambiguous), Emotion Regulation Questionnaire cognitive appraisal component (ERQ-appraisal) and emotional suppression component (ERQ-suppression).*

*Significant effect of Age Group at p < 0.0001.

*^†^Significant effect of Mood Condition at p < 0.05.*

Young adults were recruited through the University of Ottawa’s undergraduate research pool and received course credit for their participation. Older adults were recruited from the Ottawa area and received $20 for their participation. Participants provided their written informed consent and completed the study in English or French. This study was approved by the University of Ottawa Research Ethics Board (#H12-14-14).

### Stimuli for Memory Task

A set of 10 positive, 10 negative, and 10 neutral pictures were selected from a larger set of pictures published in Ack Baraly et al. (2019). The pictures were from the International Affective Picture System (Lang et al., 2008), the Geneva Affective Picture Database (Dan-Glauser and Scherer, 2011), and the internet. The pictures depicted scenes of people, objects and homes. The valence and arousal of pictures were rated on a 9-point scale using the Self Assessment Manikins from Lang et al. (2008). The three categories of pictures differed in their level of valence, and the positive and negative pictures were matched in arousal (see Table 2). The three picture categories were also matched in semantic interrelatedness. If picture categories are more interrelated and easier to organize semantically than others (e.g., positive pictures of family and love versus random neutral pictures), this can make them easier to remember. The interrelatedness of picture pairs was rated from 1 (not at all related) to 7 (extremely related), as per Talmi and McGarry (2012). In addition to the 30 target stimuli, 4 buffer images were chosen (1 positive, 1 negative, 2 neutral) to reduce effects of primacy and recency on memory.

**TABLE 2:** Mean (SD) ratings of pictures.

	Valence	Arousal	Semantic interrelatedness
Positive	2.96 (1.86)	4.67 (2.55)	3.99 (2.19)
Negative	7.68 (1.59)	4.56 (2.66)	3.88 (2.25)
Neutral	5.01 (1.16)	7.19 (2.11)	3.84 (2.08)

*Ratings and procedures are described in Ack Baraly et al. (2019). Valence ranges from 1 (happy) to 9 (unhappy) and arousal from 1 (excited) to 9 (calm). Semantic interrelatedness of picture pairs ranges from 1 (not at all related) to 7 (extremely related).*

### Mood Manipulation

#### Video Stimuli

We chose to pilot a new set of video clips from online sources rather than select from existing affective film databases (see Gilman et al., 2017), which often include scenes from well-known movies (e.g., *Bambi* or *When Harry Met Sally*). Participants’ previous exposure to movies may alter their attention (Hutchinson and Turk-Browne, 2012; Kuhl and Chun, 2014) and emotional responses (Gabert-Quillen et al., 2015). Also, movies may be heavily edited and scripted, thus reducing their affective realism (Samson et al., 2016). This may be especially true of older movies that appear outdated. As an alternative, researchers may use amateur videos from online sources because these depict real-life events and may elicit more naturalistic emotional responses than movie clips (Samson et al., 2016).

Our pilot study included 29 video clips (11 positive, 8 negative, 10 neutral). A total of 32 men and 46 women (mean age = 20.29 years) viewed a subset of the videos and rated their emotional valence and arousal on a 9-point scale (Lang et al., 2008). They also indicated whether they had seen the clip prior to the study (see Supplementary Appendix A for mean ratings for each of the 29 videos). Each video was rated by a minimum of 30 participants. From these ratings, six videos were selected for our mood-induction protocol (see Table 3 for mean ratings). Two videos were selected per valence to reduce any possible confounding effects specific to unique attributes in individual videos. One animal and one human video was selected for each of the emotional categories although the neutral videos contained only humans (due to the generally positive ratings of “neutral” animal videos). The six videos were trimmed to exactly 3 min in length using Filmora software. A description and URL for all 29 videos are listed in Supplementary Appendix B.

**TABLE 3:** Self-report ratings of videos from pilot study.

Video clip	*N*	Valence		Arousal		Familiarity (%)
				
		*M*	SD	*M*	SD	
**Positive**						
Babies laughing 1	34	2.03	1.13	4.03	2.30	26.5
Dogs and stairs 2	37	2.38	1.44	4.57	2.61	5.4
**Neutral**						
Library tour	35	5.00	1.31	7.51	2.06	0
Van Gogh tour	32	5.53	1.59	7.90	1.56	0
**Negative**						
Dog eye surgery	37	7.58	1.83	5.06	2.46	0
Huntington’s disease 2	37	7.73	1.45	5.49	2.05	0

*Valence was rated from 1 (happy) to 9 (unhappy), and arousal from 1 (excited) to 9 (calm). Familiarity refers to the percentage of participants who indicated having seen the video prior to the experiment.*

#### Mood Validation

Participants were presented with one of the six video clips to induce a positive, negative or neutral state. To validate the mood induction, participants self-reported their level of valence (unhappy to happy) and arousal (calm to excited) from 0 to 100 using the affective slider (Betella and Verschure, 2016) at baseline, after the video, and after the memory task. Participants also indicated (*yes* or *no*) whether they had seen the video prior to the study. At the end of the experiment, participants were asked whether they could guess the research hypotheses to ensure that they were not responding based on their perceived goals of the study.

### Procedures

At the start of the experiment, all participants were asked to sit for 3 min to relax. Participants then completed the first affective slider on valence and arousal (Betella and Verschure, 2016). This was followed by the Positive and Negative Affect Schedule (PANAS; Watson et al., 1988), which measures their level of positive and negative affect by indicating to what extent they currently feel each of 20 emotional adjectives (10 positive, 10 negative) on a scale from 1 (very slightly or not at all) to 5 (extremely). We then presented participants with one of the six videos randomly. They were instructed to watch the video as if they were watching a television. After the video, they once again remained seated for 3 min with no task to complete. Then they completed the second affective slider followed by the memory task.

The memory task contained three parts: an incidental encoding phase of 30 target images, an arithmetic distraction task (1 min), and a final written recall test (5 min). Participants were instructed to watch the pictures as if they were watching a television. Target pictures appeared on the screen for 4 s each in a mixed-valence random order, followed by a white inter-stimulus screen for 500 ms. Two buffer images appeared at the start and end (1 negative, 1 positive, 2 neutral) to minimize the potential effects of primary and recency. After the picture presentation, participants completed simple arithmetic calculations (e.g., “5 + 2 = ?”) during 1 min. Participants were then given 5 min to write down brief descriptions of as many pictures as they could remember. They could take up to an additional 5 min for the free recall task as needed. At the end of the memory task, participants remained seated for 3 min. Afterward, participants reported their current level of valence and arousal using the final affective slider and completed a second PANAS. At this point, participants were given a break before continuing with the remaining tasks. After the break, participants were asked to complete the MoCA, CES-D, and Future Time Perspective (FTP) scales. Two FTP scores were calculated. First, a FTP total score was calculated using the entire scale from Carstensen and Lang (1996). Second, a FTP ambiguous score was calculated using four ambiguous statements from Brothers et al. (2014) to specifically measure ambiguous time orientation. Participants also completed the Emotion Regulation Questionnaire (Gross and John, 2003), measuring both cognitive appraisal and emotional suppression. At the end of the session, we showed participants a funny dog video to minimize possible deleterious effects of the video and picture tasks, followed by a thorough debriefing. The entire session lasted up to 2 h.

### Statistical Analyses

The free recall data were scored using the same criteria described in Ack Baraly et al. (2019). Each picture was considered a correct match if the rater could identify which picture was being described. The first author (KT) scored all of the recall data for young and older adults. A second rater double scored all of the young adult data and another rater double scored the data from 70 older adults (i.e., 63% of the sample). We calculated inter-rater reliability using Pearson’s correlations for each age group and picture category separately.

Between-subject ANOVAs of Age (young, older) and Mood Condition (negative, positive, neutral) were conducted with the following dependent variables: Age, Education, CES-D, FTP-total, FTP-ambiguous, ERQ-appraisal, ERQ-suppression, or MoCA (older adults only). These variables all assess traits that should not be influenced by the mood manipulation. We expected differences between young and older adults on many if not all of these variables. The purpose of the ANOVA was to ensure that young and older adults’ reports did not change based on the mood condition (i.e., they were all randomly assigned to a mood condition so no main effect or interaction should be seen with mood condition).

We compared participants’ past familiarity with the videos by using an independent-samples non-parametric analysis (Kruskal–Wallis test) to test whether the positive, negative, or neutral videos were more familiar to participants. A non-parametric analysis was required because of the non-normality of familiarity responses in the negative and neutral conditions. This test was performed separately for the young and older adults. Only 0% to 2% of participants had previously seen the negative or neutral videos, whereas 9% of older adults and 21% of young adults had seen the positive video prior to the experiment. Therefore, for participants who viewed the positive video, we calculated the correlation between their video familiarity and self-reported emotional responses (valence, arousal, PANAS subscales) and recall (for each of the picture types).

Then, we conducted two sets of analyses on the self-reported measures of valence, arousal, and positive/negative affect (PANAS). We performed a log transformation to normalize the positive and negative PANAS scores and used these transformed data in the subsequent analyses. First, we tested whether baseline differences existed between young and older adults. To this end, we performed an independent samples *t*-test comparing Age Groups (young, older) in their self-reported valence, arousal, and PANAS scores (positive and negative). Second, to ensure that the mood manipulation was effective, a repeated measures ANOVA with Age (young, older) and Mood Condition (neutral, negative, positive) as between-subjects factors and Time (baseline, post-video, post-memory) as a within-subjects factor was performed on the ratings of valence and arousal. No differences between mood conditions should be observed at baseline although significant differences in valence and arousal should appear at the second and third time points. Similarly, no differences in positive and negative affect (measured with the PANAS) should exist between the mood conditions at baseline, although differences should appear after the memory task.

Then, a 2 × 3 × 3 mixed ANOVA was performed with Age (young, older) and Mood (positive, negative, neutral) as the between-subjects factors and Picture Type (positive, negative, neutral) as the within-subjects factor, on the total number of pictures correctly recalled.^3^ We computed additional mixed ANOVAs to follow-up on significant interactions. Crucially, we tested whether an interaction existed between Mood and Picture Type because the direction of the memory bias (positive or negative) should vary between the mood conditions. This also allowed us to test whether the effects of mood on recall were the same in both age groups.

Finally, we calculated a positivity of recall score as per Barber et al. (2016): the number of correctly recalled negative pictures was subtracted from the correctly recalled positive pictures, the sum being divided by the total number of pictures recalled [(positive – negative)/(positive + negative + neutral)]. This allowed us to directly test the magnitude of the positive bias as a function of self-reported measures. We calculated the Pearson’s correlations between positivity of recall and valence (3 time points), arousal (3 time points), PANAS positive and negative affect (baseline, post-memory task), FTP-Total, FTP-Ambiguous, ERQ-Appraisal, ERQ-Suppression, and CES-D. Only one variable significantly correlated with positivity of recall so there was no need to calculate a regression model with multiple linear regression.

## Results

### Data Cleaning and Screening

The data were screened for outliers and violations of normality using the Kurtosis and Skewness values in SPSS. The demographic and questionnaire data were normally distributed with no outliers, except for age and education which were both positively skewed in young adults. This is expected because university students are predominantly of the same age and education. Free recall data were also normally distributed with no outliers. The distribution of scores for valence and arousal were normal (Kurtosis and Skewness values 1.5 or lower). However, the interquartile range of arousal scores for older adults was very small, leading to a dozen outlying scores identified in SPSS. Next, we calculated the *z*-scores for each arousal time point for older adults, and all scores were below 3. Therefore, all data were retained. The PANAS positive subscale had normally distributed data with no outliers, but the negative subscale (both time points) was positively skewed. For consistency, we performed a log transformation for both positive and negative subscales to normalize the data. There were no violations in sphericity in any of the analyses.

There was also a number of self-reported valence and arousal responses missing in the sample. Missing data occurred in 14 young and 13 older adults where there was at least one response of valence or arousal missing. Missing data for valence and arousal totaled 2.27% in young adults and 2.85% in older adults. These scales serve as a behavioral validation of the mood manipulation technique and for that reason, it did not seem appropriate to replace the missing data (e.g., with the mean score). As a result, participants with missing data were removed from the analysis of valence and arousal.

### Inter-Rater Reliability

Pearson’s correlations between the two raters for young adults were high for negative (*r* = 0.95), positive (*r* = 0.96), and neutral (*r* = 0.93) pictures. The correlations between the two raters for older adults was also high for negative (*r* = 0.93), positive (*r* = 0.85), and neutral (*r* = 0.94) pictures. The primary rater (KT) checked all disagreements to ensure accuracy and consistency across the ratings.

### Demographics and Questionnaire Data

Means differed significantly between age groups for Education [*F*(1, 257) = 264.50, *p* < 0.0001], CES-D [*F*(1, 255) = 67.22, *p* < 0.0001], FTP-total [*F*(1, 256) = 75.32, *p* < 0.0001], FTP-ambiguous [*F*(1, 256) = 15.95, *p* < 0.0001], and ERQ-suppression [*F*(1, 256) = 17.84, *p* < 0.0001]. Young adults had fewer years of education and reported more depressive symptoms than older adults. They also viewed their future as open-ended yet ambiguous, and they suppressed their emotions more than older adults, as shown in Table 1.

There was also a main effect of Mood Condition for the MoCA in older adults [*F*(2, 109) = 3.94, *p* = 0.022]. Older adults who viewed the negative video performed lower on the MoCA than those who viewed the neutral video (Bonferroni-corrected *p* = 0.035). The MoCA was not administered to young adults; therefore, it was not inputted as a covariate in the original analysis. No other factor was influenced by Mood Condition.

### Video Familiarity

Young and older adults were more likely to have previously seen the positive video than the negative or neutral video [young: Kruskal–Wallis *H*(2) = 18.99, *p* < 0.0001; older: Kruskal–Wallis *H*(2) = 6.92, *p* = 0.031; Table 4]. Familiarity with the positive video did not significantly correlate with any of the self-reported emotional responses or recall, so it was not considered in subsequent analyses.

**TABLE 4:** Mean (SD) self-reported valence, arousal, and positive and negative affect by age group and mood condition.

		Young adults			Older adults		
			
	Mood condition						
		
		Neutral	Negative	Positive	Neutral	Negative	Positive
	*Total N*	55	48	44	38	39	34
	*Familiarity*	0%	2%	21%	0%	0%	9%
Valence	*n*	51	44	43	37	39	32
	Baseline	64.29 (17.24)	69.05 (16.05)	69.37 (17.82)	80.95 (14.95)	74.49 (20.09)	76.38 (17.38)
	Post-video	55.02 (16.66)	32.91 (19.31)	77.19 (16.58)	64.59 (22.22)	32.92 (21.60)	79.13 (26.77)
	Post-memory	54.02 (17.37)	49.98 (16.43)	55.42 (19.79)	68.03 (18.96)	56.82 (21.25)	58.44 (18.25)
Arousal	*n*	50	44	43	37	36	27
	Baseline	47.86 (20.85)	44.39 (20.79)	45.40 (20.56)	49.78 (24.12)	55.58 (17.10)	49.48 (22.44)
	Post-video	37.64 (19.86)	42.59 (18.54)	51.16 (21.32)	47.89 (20.98)	51.56 (19.41)	54.70 (32.81)
	Post-memory	44.54 (19.22)	45.70 (16.59)	41.98 (19.40)	57.59 (22.05)	50.56 (15.85)	53.37 (18.18)
PANAS positive	*n*	55	48	43	38	39	34
	Baseline	29.87 (7.40)	29.35 (6.22)	28.47 (7.47)	34.21 (7.49)	33.49 (6.75)	34.15 (8.04)
	Post-Memory	26.93 (8.27)	27.23 (7.72)	25.70 (8.26)	33.55 (7.56)	31.54 (7.78)	33.79 (7.90)
PANAS negative	*n*	55	48	43	38	39	34
	Baseline	16.58 (6.88)	15.44 (5.04)	14.88 (6.00)	12.11 (3.14)	11.28 (1.91)	12.32 (3.08)
	Post-memory	15.16 (5.55)	15.81 (5.93)	16.02 (7.13)	12.29 (3.14)	12.10 (2.49)	13.18 (4.54)

*Familiarity represents the percentage of participants who had seen the video before the study. Valence was rated from 0 (unhappy) to 100 (happy) and arousal was rated from 0 (calm) to 100 (excited). PANAS, Positive and Negative Affect Schedule.*

### Self-Reported Emotional Responses

#### Baseline

At baseline, older adults reported more positive valence [*t*(248) = 4.53, *p* < 0.0001] and positive affect [*t*(255) = 5.15, *p* < 0.0001], and less negative affect [*t*(242) = 7.42, *p* < 0.0001], compared to young adults (Table 4). Older adults also seemed to report higher levels of arousal [*t*(244) = 2.08, *p* = 0.038], but this difference was not significant when controlling for multiple *t*-tests (alpha 0.05/4 = 0.0125).

#### Valence (Scale From 0 to 100)

Self-reported valence differed significantly between the two Age Groups [*F*(1, 240) = 15.63, *p* < 0.0001, $\eta_{p}^{2}$ = 0.06]: Older adults reported higher (i.e., more positive) levels of valence than young adults (mean valence of 65.29 vs. 58.48, respectively). There was also a main effect of Mood Condition [*F*(2, 240) = 29.38, *p* < 0.0001, $\eta_{p}^{2}$ = 0.20], with responses being lower in the negative condition (*M* = 52.69), higher in the neutral condition (*M* = 64.48), and highest in the positive condition (*M* = 69.32). These main effects were characterized by an Age Group × Mood Condition interaction [*F*(2, 240) = 3.07, *p* = 0.048, $\eta_{p}^{2}$ = 0.03]. Whereas young adults reported different levels of valence in each condition (*p*s < 0.05), older adults reported lower valence in the negative condition only (*p*s < 0.0001) and equally high valence in both positive and neutral conditions (*p* > 0.90). This suggests that participants generally responded as expected to the video manipulation, with the exception of older adults responding positively to the neutral condition. In addition, there was a main effect of Time [*F*(2, 480) = 81.13, *p* < 0.0001, $\eta_{p}^{2}$ = 0.25] because mean valence was significantly different between baseline and post-video (*p* < 0.0001) and between baseline and post-memory task (*p* < 0.0001), but not between post-video and post-memory task (*p* = 0.71). This was influenced by a Time × Mood Condition interaction [*F*(4, 480) = 52.30, *p* < 0.0001, $\eta_{p}^{2}$ = 0.30]. At baseline, valence was similar in all three conditions (*p*s > 0.40). After the video, participants reported lower valence in the negative condition, higher valence in the neutral condition, and even higher valence in the positive condition (*p*s < 0.0001). After the memory task, valence was once again similar in all three conditions (*p*s > 0.05). Participants generally started the experiment in a relatively good mood at baseline, then after viewing the video their mood changed based on the condition they were assigned to, and by the end of the memory task most participants were in a more neutral mood. The three-way interaction of Age Group × Mood Condition × Time was not significant.

#### Arousal (Scale From 0 to 100)

Self-reported arousal differed significantly between the two Age Groups [*F*(1, 231) = 14.31, *p* < 0.0001, $\eta_{p}^{2}$ = 0.058]: On average, older adults reported higher levels of arousal than young adults (*M* = 52.25 vs. 44.52, respectively). There was also a Mood Condition × Time interaction [*F*(2, 462) = 3.81, *p* = 0.005, $\eta_{p}^{2}$ = 0.032]. At baseline, arousal was similar in all three conditions (*p*s > 0.70). Then, participants reported significantly higher arousal after the positive video than after the neutral video (*p* = 0.003), yet they reported similar levels of arousal after the negative and neutral videos (*p* = 0.07), and negative and positive videos (*p* = 0.11). After the memory task, arousal was once again similar in all three conditions (*p*s > 0.40). This suggests that the positive video led to the greatest increase in self-reported arousal, when averaging young and older adult responses together.

#### Positive Affect (Positive and Negative Affect Schedule Positive Subscale)

Self-reported positive affect differed significantly between the two Age Groups [*F*(1, 251) = 34.56, *p* < 0.001, $\eta_{p}^{2}$ = 0.121]. On average, older adults reported higher positive affect than young adults (*M* = 33.43 vs. 27.98, respectively). Positive affect also differed significantly between the two Times [*F*(1, 251) = 43.73, *p* < 0.001, $\eta_{p}^{2}$ = 0.148]. In general, positive affect was higher at baseline and lower by the end of the memory task (*M* = 31.30 vs. 29.37, respectively). There was also an Age Group × Time interaction [*F*(1, 251) = 12.55, *p* < 0.001, $\eta_{p}^{2}$ = 0.048]. However, *post hoc* paired samples *t*-tests showed that both Age Groups reported significantly higher positive affect at baseline than at the end of the memory task [Young adults: *t*(145) = 6.88, *p* < 0.001; Older adults: *t*(110) = 2.69, *p* = 0.009]. Descriptively, the mean difference between time points was greater in young than in older adults, suggesting that young adults’ positive affect decreased more.

#### Negative Affect (Positive and Negative Affect Schedule Negative Subscale)

Self-reported negative affect differed significantly between the two Age Groups [*F*(1, 251) = 40.89, *p* < 0.0001, $\eta_{p}^{2}$ = 0.140]. On average, young adults reported higher negative affect than older adults (*M* = 15.67 vs. 12.19, respectively). There was also a significant Mood Condition × Time interaction [*F*(2, 251) = 3.14, *p* = 0.045, $\eta_{p}^{2}$ = 0.024]. *Post hoc* paired samples *t*-tests compared the Times for each Mood Condition separately, using a Bonferroni-corrected alpha of 0.017 (0.05 alpha/3 contrasts). Using the corrected alpha, there was no significant difference between baseline and post-memory task for the negative [*t*(86) = 1.25, *p* = 0.216], positive [*t*(76) = 1.83, *p* = 0.071], or neutral conditions [*t*(92) = 1.97, *p* = 0.051].

### Memory

**FIGURE 1:**
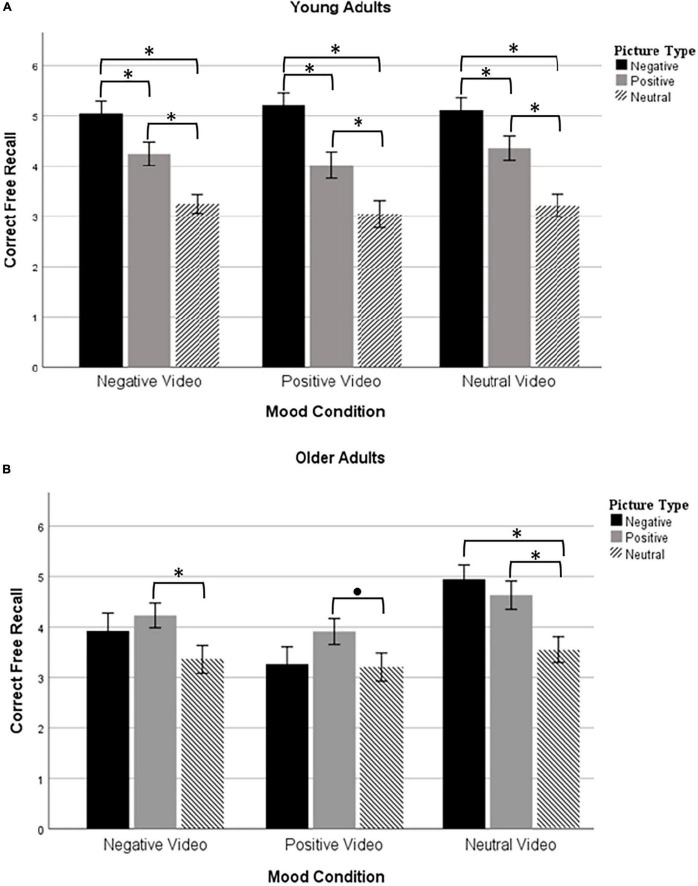
Mean correct recall in young adults **(A)** and older adults **(B)** after watching either a negative, positive, or neutral video. *p < 0.05 and ^•^*p* < 0.10 after Bonferroni adjustment for multiple comparisons.

The mixed ANOVA revealed a main effect of Mood Condition [*F*(2, 252) = 3.60, *p* = 0.029, $\eta_{p}^{2}$ = 0.028] and Picture Type [*F*(2, 504) = 60.59, *p* < 0.0001, $\eta_{p}^{2}$ = 0.194], and an Age Group × Picture Type interaction [*F*(2, 504) = 16.25, *p* < 0.0001, $\eta_{p}^{2}$ = 0.061; see Figure 1]. The three-way interaction between Age Group × Mood Condition × Picture Type was marginal [*F*(4, 504) = 2.05, *p* = 0.087, $\eta {}_{p}^{2}$ = 0.016].

First, participants in the neutral mood condition recalled more pictures in total than those in the positive mood condition (*M* = 4.31 vs. 3.78, respectively; Bonferroni-corrected *p* = 0.048). There were no differences in overall recall between the other mood conditions (negative vs. neutral conditions: *p* > 0.50; negative vs. positive conditions: *p* > 0.80). In regard to Picture Type, negative pictures were recalled more often than positive [*t*(257) = 3.28; *p* = 0.001] or neutral pictures [*t*(257) = 10.90; *p* < 0.0001], and positive pictures were recalled more often than neutral pictures [*t*(257) = 3.28; *p* < 0.0001]. This pattern of recall (negative pictures > positive pictures > neutral pictures) appeared in young adults, but was not present in older adults (Age Group × Picture Type interaction). On the contrary, older adults seemed to recall negative and positive pictures equally well [*t*(110) = 1.06; *p* = 0.292], both of which were better recalled than neutral pictures [negative vs. neutral: *t*(110) = 3.73; *p* < 0.0001; positive vs. neutral: *t*(110) = 5.44; *p* < 0.0001].

The marginal three-way interaction [Age Group × Mood Condition × Picture Type; *F*(4, 504) = 2.05, *p* = 0.087, $\eta_{p}^{2}$ = 0.016] was central to our main hypotheses, so we performed follow-up analyses separated by Age Group despite the interaction being toward significance. In young adults, a mixed ANOVA revealed a main effect of Picture Type [*F*(2, 288) = 67.71, *p* < 0.001, $\eta_{p}^{2}$ = 0.32], but no main effect of Mood Condition [*F*(2, 144) = 0.17, *p* = 0.846, $\eta_{p}^{2}$ = 0.002] and no Mood Condition × Picture Type interaction [*F*(4, 288) = 0.39, *p* = 0.819, $\eta_{p}^{2}$ = 0.005]. As described above, young adults showed a persistent negativity bias. They recalled negative pictures more often than positive (*p* < 0.001) or neutral pictures (*p* < 0.001), and positive pictures more often than neutral pictures (*p* < 0.001). A different pattern emerged in older adults. In older adults, a mixed ANOVA revealed a main effect of Mood Condition [*F*(2, 108) = 4.01, *p* = 0.021, $\eta_{p}^{2}$ = 0.069], a main effect of Picture Type [*F*(2, 216) = 13.61, *p* < 0.001, $\eta_{p}^{2}$ = 0.112], and a Mood Condition × Picture Type interaction [*F*(4, 216) = 2.54, *p* = 0.041, $\eta_{p}^{2}$ = 0.045]. The main effect of Picture Type was significant in older adults in the negative mood condition [*F*(2, 76) = 4.24, *p* = 0.018, $\eta_{p}^{2}$ = 0.100] and the neutral mood condition [*F*(2, 75) = 13.19, *p* < 0.001, $\eta_{p}^{2}$ = 0.263], but did not reach significance for those in the positive mood condition [*F*(2, 66) = 2.77, *p* = 0.070, $\eta_{p}^{2}$ = 0.077]. Older adults in the negative mood condition recalled more positive than neutral pictures (*p* = 0.008). In the neutral condition, they recalled more positive (*p* = 0.002) and negative pictures (*p* < 0.001) than neutral pictures. The difference was not significant between positive and neutral pictures in the positive mood condition (*p* = 0.070). In no conditions did older adults recall positive pictures more than negative pictures. These main effects and interaction were no longer significant when adding MoCA scores as a covariate in the analysis (as recommended by a reviewer in light of baseline differences in MoCA across Mood Condition).

**FIGURE 2:**
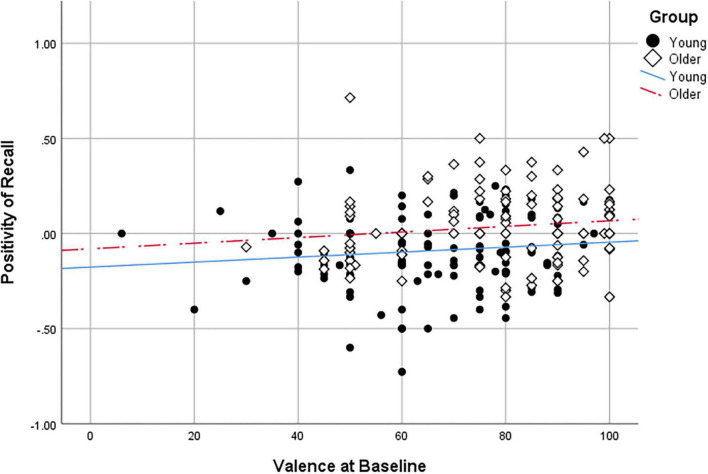
Positivity of recall scores as a function of valence at baseline in young adults and older adults.

**FIGURE 3:**
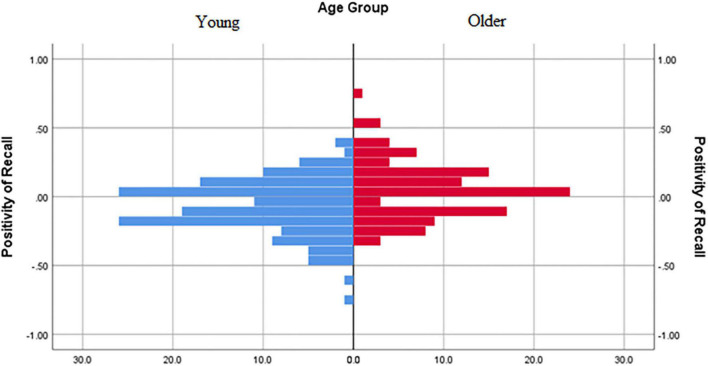
Frequency distribution of positivity of recall for young adults and older adults. Positive values reflect a positive memory bias and negative values reflect a negative memory bias.

We examined more specifically whether any of the self-reported measures significantly predicted emotional memory biases using the computed positivity of recall score (following the methods outlined in Barber et al., 2016). Data from one older adult were removed because the positivity of recall score was an extreme (positive) outlier. Only valence at baseline significantly correlated (*r* = 0.234, *p* < 0.0001) with positivity of recall when using a Holm-Bonferroni corrected alpha (see Figure 2). There were no significant correlations with other valence timepoints (post-video, post-memory task), arousal, PANAS positive, PANAS negative, FTP-Total, FTP-Ambiguous, ERQ-Appraisal, ERQ-Suppression, or CES-D (see correlation table in Supplementary Appendix C). The frequency distribution of the positivity of recall scores for young and older adults is shown in Figure 3. As confirmed in the ANOVA, young adults displayed a negativity bias more often than older adults. Yet, it is interesting to note that both groups showed a range of positive and negative memory biases.

## Discussion

Aging is associated with some improvements in mood, yet the impact of these changes on the age-related positivity effect in memory is not often explored. In the current study, participants completed a mood induction protocol designed to promote either a negative, positive, or neutral mood. This was followed by a free recall picture memory task. The effectiveness of the mood induction protocol was assessed with self-reported behavioral measures (i.e., valence, arousal, and positive and negative affect; PANAS). Overall, the mood induction protocol was successful in changing people’s self-reported valence, yet it had little impact on self-reported arousal. Young adults showed a consistent negative memory bias. Older adults did not show a negativity bias in memory, but rather a memory advantage for positive over neutral pictures which varied somewhat by mood condition.

### Age Differences in Affect, Time Perspective, and Emotion Regulation

We argued that the positivity effect in aging might result, at least in part, from young and older adults differing in their mood at the start of an experiment. Indeed, young and older adults reported different levels of valence and affect. At baseline, older adults were more positive (valence and PANAS positive scales) and less negative (PANAS negative scale) than young adults. Furthermore, in comparison to young adults, older adults reported fewer depressive symptoms over the past week. Similar findings are reported in previous papers on emotional memory and aging (e.g., Charles et al., 2003; Mather and Knight, 2005; Emery and Hess, 2008, 2011; Fernandes et al., 2008; Tomaszczyk et al., 2008; Mickley and Kensinger, 2009; Fung et al., 2010). These results are consistent with lifespan studies showing linear increases in positive affect and decreases in negative affect during adulthood and into older age (Kunzmann et al., 2000; Gana et al., 2015).

We also found that young adults viewed their future as more open-ended, albeit ambiguous, than older adults. This is expected because the average young adult has more time left in life than the average older adult, consistent with Socioemotional Selectivity Theory (Carstensen et al., 1999; Reed and Carstensen, 2012). However, this may also lead young adults to be uncertain of their future and view it as more ambiguous, especially for university students who have not yet started their career and post-academic life. We also found that young adults suppressed their emotions more than older adults, as indicated by higher scores on the Emotion Regulation Questionnaire (suppression component). This suggests that young adults adopt more maladaptive emotion regulation strategies than older adults. They may be more likely to suppress their feelings when dealing with stressful situations, which can lead them to also suppress feelings of positive emotions too (Gross and John, 2003). In fact, emotion suppression might have the opposite desired effect by leading them to feel even more negative emotions than those who do not use suppression (Gross and John, 2003). This is coherent with the observation of baseline differences in affect between the two groups. Overall, these results show that older adults were in a more positive mood at the start of the experiment, were more likely to engage in adaptive emotion regulation strategies in their daily life, and viewed their futures as more restrictive than young adults. However, age differences in affect, time perspective and emotion regulation did not predict emotional memory biases. More direct evidence is still needed (Chukwuorji and Allard, 2022).

### Effectiveness of the Mood Induction Protocol

A mood induction protocol helps exert experimental control and reduces the noise of interindividual differences when examining the interaction between mood and memory. Overall, the mood manipulation temporarily altered young and older adults’ emotional state, as measured by self-reported valence (i.e., degree of pleasantness). There were no differences in valence reported across the three mood conditions at baseline. The videos induced the desired emotional response in young adults. They reported more of an unpleasant state after a negative video, a pleasant state after a positive video, and a neutral state after a neutral video. On the other hand, older adults reacted positively to both positive and neutral videos, and showed a significant unpleasant response to the negative videos. This is not surprising given that other authors have found that older adults respond more positively to neutral film clips (Fernández-Aguilar et al., 2018) and pictures (van Reekum et al., 2011), compared to young adults. In fact, in the present study, older adults reported higher (more positive) valence overall, compared to young adults. Once again, this supports our general findings that older adults reported higher positive affect and lower negative affect from the start of the experiment. Nonetheless, the video clips in the current study successfully elicited negative and positive emotions in both young and older adults, despite the neutral (control) condition being positively biased in older adults. This is an important strength in the current study because previous work has shown that eliciting positive emotions can be challenging (Beaudreau et al., 2009; Fernández-Aguilar et al., 2018).

Interestingly, only the positive videos induced higher arousal compared to the neutral videos; arousal was otherwise equal between neutral and negative videos. The positive videos might have led to higher arousal because they depicted scenes of babies laughing and funny dogs, clips that were selected to target humor and amusement. These clips would land higher on the dimension of activation (Russell, 2003) than would the negative videos which targeted scenes of sadness and mild distress. We intentionally chose low activation negative videos because we believed sadness and distress would better reflect the sources of low mood in young adults (versus more intense negative emotions of fear or anger). Once again, older adults reported higher arousal levels than young adults when considering all conditions and time points. This was unexpected because young adults are more likely to experience high arousal during positive and negative experiences, whereas older adults are more likely to experience lower arousal during positive experiences (Keil and Freund, 2009; Fernández-Aguilar et al., 2018). As has been pointed out elsewhere (Fernández-Aguilar et al., 2018), little work has been done to explore differences between discrete positive emotions in emotion-eliciting film sets, and comparisons between different age groups is even more rare. Some of the variability across studies could result from different discrete emotions being elicited, which can be considered in future studies.

Despite the mood manipulation successfully eliciting negative and positive emotional responses in young and older adults, these differences did not last until the end of the experiment. By the end of the memory task, both age groups returned back to baseline levels in self-reported valence and arousal. This means that the emotions elicited in response to the video did not last until the end of the memory task. This shift back to baseline levels was also observed in Knight et al. (2002) using the Depressive Adjective Checklist as a validation for their sad mood induction. This may be more likely in studies involving an emotional memory task because viewing emotional stimuli can interfere with the emotion elicited by the mood induction protocol.

### Mood Induction and Memory

The main purpose of this study was to test whether emotional memory biases in young and older adults could be explained by their moods. Contrary to our predictions, young adults consistently showed a negative memory bias regardless of the mood manipulation. That is, their memory was always greatest for negative pictures, lower for positive pictures, and lower still for neutral pictures. A negativity bias in young adults is commonly reported in the literature (Baumeister et al., 2001; Reed et al., 2014; Carstensen and DeLiema, 2018). This was not the case for older adults, who had similar recall rates for positive and negative pictures. Equal memory for positive and negative information in aging has also been shown in previous work (Kensinger et al., 2007).

Therefore, in neither age group was there an apparent mood congruence effect. Valence at baseline significantly correlated with the positivity of recall scores, but this was a weak correlation (*r* < 0.30). Contrary to our predictions, follow-up analyses to the marginal 3-way interaction (Age Group × Mood Condition × Picture Type) suggest there may have even been mood incongruent effects. In the neutral mood condition, older adults’ memory was greater for positive and negative pictures than for neutral pictures, with no advantage of one emotion over the other. In the negative mood condition, older adults recalled more positive than neutral pictures, and equal amounts of negative and neutral pictures. However, the advantage of positive over neutral pictures did not reach significance in the positive mood condition. Mood-incongruence may serve to regulate emotions (Sedikides, 1994; Forgas and Ciarrochi, 2002), which could explain why the advantage of positive over neutral pictures was most apparent in older adults in the negative mood condition. Indeed, previous work on attentional gaze showed that older adults were more likely to demonstrate mood-incongruent gaze toward faces when unhappy (Isaacowitz et al., 2008). It is also noteworthy that older adults in the neutral condition did not show a memory advantage for positive over negative pictures. We might have expected a positivity bias to appear because this is a control condition. Upon closer look (Table 4), we can see that older adults’ self-reported valence decreased from 81 at baseline to 65 after the neutral video. Despite them reporting a positive mood in absolute terms, there was a large relative decrease in mood which might explain in part the absence of a positivity bias.

Taken together, the results of this study do not support a mood-congruent memory hypothesis of the positivity effect in aging. This is contrary to previous work (Knight et al., 2002) which reported some mood-congruent memory effects using a combined Velten and music induction technique. In Knight et al., older adults recalled fewer positive words when they were induced into a sad mood than when induced into a neutral mood. The authors did not compare the relative difference in recall between positive and negative pictures within a mood condition, which would have allowed them to more directly test the positivity effect as defined by Reed et al. (2014). Upon a closer look at their results (Knight et al., 2002), older adults remembered positive and negative words equally well on an immediate recall test, revealing that there was no strong mood-congruent memory effect. On the delayed recall test, however, older adults seemed to show a negativity bias in the sad mood condition. It would be interesting to explore this in future work to see whether mood-congruent memory effects appear in older adults after longer test delays.

The current results support the view that the positivity effect in aging is the result of a negativity bias that fades with age (Carstensen and DeLiema, 2018). The negativity bias was consistent across all three mood conditions in young adults, and this shifted toward no negativity bias (or even a slight positivity preference) in older adults. This shift in emotional memory bias was further illustrated in the frequency distributions of the positivity of recall scores. These frequency distributions also showed that many young adults had a positivity bias and many older adults had a negativity bias. Although the general means might shift toward more positivity with age, many individual differences exist within each age group. In the present study, these differences were not explained by mood, nor were they explained by future time perspective, as would be predicted by Socioemotional Selectivity Theory (Reed et al., 2014; Carstensen and DeLiema, 2018).

In older adults, there also seemed to be some costs to viewing an emotional video at the start of the experiment. Older adults who viewed a negative video later performed worse on a measure of general cognitive ability (i.e., MoCA) than those who had viewed a neutral video at the start of the experiment. This was despite them reporting similar levels of valence and arousal at the end of the experiment. Total picture recall was also lower (for both age groups) after viewing a positive video than after viewing a neutral video. Older adults have been shown to pay more attention to positive stimuli than to negative stimuli (Isaacowitz et al., 2009). It is possible that they were more engaged and invested in the positive video versus the neutral video while it was playing. This could have made it harder for them to disengage their attention and thoughts from the video after it stopped playing, to task switch and focus on the subsequent picture task. In light of the incidental encoding instructions, these older adults might have continued thinking about the positive video because they did not have to explicitly memorize or act on the pictures being shown. For the time being, this interpretation remains speculative until more work explores these hypotheses directly. Overall, in the present study, both emotional mood induction conditions seemed to incur a cognitive cost to older adults. However, this does not rule out the possibility that true differences in general cognitive ability existed at baseline between the different groups of older adults. Care should be taken when using mood induction techniques with older populations as these emotional manipulations might alter other cognitive functions.

## Conclusion

In the present paper, young adults showed a persistent negative memory bias in all mood conditions. Older adults showed no explicit positivity bias and recalled positive and negative pictures equally well. The mood manipulation somewhat affected older adults’ emotional memory as there was some indication of mood incongruence which might serve to regulate emotions. These results lend limited support to the mood-congruent hypothesis and socioemotional selectivity theory for the positivity effect in aging. Mood may influence in part older adults’ emotional memory and should be considered in studies on aging and memory. The robust age group differences support the view that the positivity effect in aging is the result of a negativity bias that fades with age (Carstensen and DeLiema, 2018).

## Data Availability Statement

The raw data supporting the conclusions of this article will be made available by the authors, without undue reservation. Details on how to access the picture stimuli from the experiments can be found at https://socialsciences.uottawa.ca/neuropsychology/publications.

## Ethics Statement

The studies involving human participants were reviewed and approved by University of Ottawa Research Ethics Board. The participants provided their written informed consent to participate in this study.

## Author Contributions

KT, PH, and PD designed the study and analysis plan. KT, CK, and VT prepared the experimental manipulation, piloted the stimuli and conducted the experiment. All authors contributed to the data cleaning, analysis, and write-up.

## Conflict of Interest

The authors declare that the research was conducted in the absence of any commercial or financial relationships that could be construed as a potential conflict of interest.

## Publisher’s Note

All claims expressed in this article are solely those of the authors and do not necessarily represent those of their affiliated organizations, or those of the publisher, the editors and the reviewers. Any product that may be evaluated in this article, or claim that may be made by its manufacturer, is not guaranteed or endorsed by the publisher.

## Footnotes

1 We also collected participant heart rate variability and cardiac impedance throughout the experiment, however, the results were inconclusive. These physiological measures did not vary as a function of mood manipulation so we report only on the self-reported ratings in this article.

2 The initial target number of participants was 140 per age group to obtain 0.90 power (calculated *a priori*), but we subsequently lowered the power requirement due to limited recruitment.

3 We did not include any of the demographic or questionnaire responses as covariates in this analysis because there was no main effect or interaction by Mood Condition. There is insufficient statistical power to include all variables with age-group differences in the mixed ANOVA. If we include a few key variables as covariates, CES-D and the two time perspective scales (because these test hypotheses of mood congruence and SST), the results of the analysis remain the same. If anything, their inclusion brings the trending effects closer to significance. For consistency and simplicity, we do not present these covariates in the ANOVA.
