# Supplementary material for: Disentangling Aging and Mood Effects on Emotional Memory
Source: Front Behav Neurosci. 2022 Aug 26;16:944363. doi: 10.3389/fnbeh.2022.944363 (PMC9462378; doi:10.3389/fnbeh.2022.944363)
Supplement: Supplementary file 1 [file Data_Sheet_1.docx]

## Disentangling aging and mood effects on emotional memory

## Supplementary material

Kylee Tamera^1,2,3^, Courtney Kannampuzha^1^, Viviane Ta^1^, Pascal Hot^2,3^, & Patrick S. R. Davidson^1^

*^1^School of Psychology, University of Ottawa
^2^ Univ. Savoie Mont Blanc, LPNC, 73000 Chambéry, France*

*^3^Institut Universitaire de France, France*

Appendix A: Self-reported ratings for all 29 video clips piloted

| Video clip | *N* | Valence | | Arousal | | Familiarity (%) |
| --- | --- | --- | --- | --- | --- | --- |
|  |  | *M* | *SD* | *M* | *SD* |  |
| **Positive** |  |  |  |  |  |  |
| Dogs and stairs 1 | 36 | 1.97 | 1.18 | 3.53 | 1.89 | 19.4 |
| Babies laughing 1† | 34 | 2.03 | 1.13 | 4.03 | 2.30 | 26.5 |
| Babies laughing 2 | 40 | 2.05 | 1.38 | 3.40 | 1.93 | 42.5 |
| Dogs and stairs 2† | 37 | 2.38 | 1.44 | 4.57 | 2.61 | 5.4 |
| Presto | 36 | 2.50 | 1.36 | 3.86 | 2.00 | 8.3 |
| Babies and dogs | 35 | 2.57 | 1.09 | 4.80 | 2.18 | 0 |
| Baby dancing | 34 | 2.76 | 1.18 | 4.71 | 1.98 | 23.5 |
| Partly Cloudy | 33 | 2.91 | 1.89 | 4.30 | 2.08 | 21.2 |
| Child scared of monsters | 36 | 3.06 | 1.43 | 5.44 | 2.10 | 22.2 |
| Baby saying no | 34 | 3.41 | 1.31 | 5.35 | 2.19 | 8.8 |
| Baby with hiccups | 32 | 3.72 | 1.73 | 5.41 | 2.21 | 3.1 |
|  |  |  |  |  |  |  |
| **Neutral** |  |  |  |  |  |  |
| Beaver | 34 | 3.44 | 1.78 | 7.15 | 2.00 | 0 |
| Turtle | 35 | 4.06 | 1.54 | 6.18 | 2.55 | 2.9 |
| Ducks | 36 | 4.42 | 1.13 | 7.64 | 2.10 | 0 |
| Dog competition | 36 | 4.50 | 1.46 | 6.75 | 2.41 | 0 |
| Interview | 36 | 4.50 | 1.11 | 5.33 | 2.23 | 0 |
| Fish | 36 | 4.67 | 1.22 | 5.56 | 2.48 | 2.8 |
| Library tour† | 35 | 5.00 | 1.31 | 7.51 | 2.06 | 0 |
| Dali museum tour | 32 | 5.28 | 1.46 | 8.31 | 1.23 | 0 |
| Hannah and her sisters | 37 | 5.32 | 1.00 | 6.73 | 1.87 | 2.7 |
| Van Gogh tour† | 32 | 5.53 | 1.59 | 7.90 | 1.56 | 0 |
|  |  |  |  |  |  |  |
| **Negative** |  |  |  |  |  |  |
| StoryCorps 1 | 31 | 6.23 | 2.55 | 4.78 | 2.64 | 3.2 |
| StoryCorps 2 | 37 | 6.84 | 1.83 | 5.43 | 2.12 | 0 |
| Children with cancer | 35 | 7.03 | 1.36 | 5.89 | 1.84 | 0 |
| Dog with lesions | 39 | 7.30 | 1.71 | 5.37 | 2.08 | 0 |
| Huntington’s disease 1 | 34 | 7.47 | 1.19 | 5.47 | 2.33 | 0 |
| Dog eye surgery† | 37 | 7.58 | 1.83 | 5.06 | 2.46 | 0 |
| Huntington’s disease 2† | 37 | 7.73 | 1.45 | 5.49 | 2.05 | 0 |
| Child with leukemia | 37 | 7.78 | 1.20 | 5.46 | 2.16 | 0 |

*Note.* Valence was rated from 1 (happy) to 9 (unhappy), and arousal from 1 (excited) to 9 (calm). Familiarity refers to the percentage of participants who indicated having seen the video prior to the experiment. ^†^Videos selected for the mood-induction protocol.

Appendix B: Description of all 29 video clips piloted

| Title | Length(min) | Target mood | Description | URL |  |
| --- | --- | --- | --- | --- | --- |
| Babies and dogs | 4:06 | Positive | Compilation video of dogs or puppies playing with babies and consists of happy background music. | https://www.youtube.com/watch?v=p336IIjZCl8 |  |
| Babies laughing 1† | 2:57 | Positive | Compilation video of babies laughing. | https://www.youtube.com/watch?v=L49VXZwfup8 |  |
| Babies laughing 2 | 3:55 | Positive | Compilation video of babies laughing. | https://www.youtube.com/watch?v=L49VXZwfup8; https://www.youtube.com/watch?v=RK4x3Snzfo0; https://www.youtube.com/watch?v=dmVzCf_G1-k |  |
| Dogs and stairs 2† | 3:02 | Positive | Compilation video of dogs trying to walk on stairs and consists of happy background music. | https://www.youtube.com/watch?v=gghfoRKVPCo |  |
| Baby dancing | 1:38 | Positive | Baby watches and dances to the song “Single ladies” by Beyonce. | https://www.youtube.com/watch?v=DnjHMtUjIvQ |  |
| Baby saying  “no no no” | 0:30 | Positive | Baby in a bathtub. Parents asks the baby a question and the baby responds with “no”. | https://www.youtube.com/watch?v=OSPGOz2K0-U |  |
| Baby with hiccups | 1:00 | Positive | Two babies sitting side by side. One baby hiccups and the other baby laughs. | https://www.youtube.com/watch?v=z5-mOiQUcrg |  |
| Child scared of monsters | 0:42 | Positive | A child tells her mother what she will do if she sees a monster. | https://www.youtube.com/watch?v=OSPGOz2K0-U |  |
| Dogs and stairs 1 | 4:03 | Positive | Compilation video of dogs trying to walk on stairs and consists of happy background music. | https://www.youtube.com/watch?v=gghfoRKVPCo | |
| Partly Cloudy | 5:00 | Positive | A cloud creates different animals for a stork to deliver. In the end, the cloud and the stork become friends. | https://www.youtube.com/watch?v=7DmLkugdh9s | |
| Presto | 4:31 | Positive | Magician performs on a stage and tries to get a rabbit to cooperate who ends up playing tricks on the magician. | https://www.youtube.com/watch?v=D4Dnm6dkOVI | |
| Children with cancer | 3:17 | Negative | Various stories of children with cancer. Video is in black and white. | https://www.youtube.com/watch?v=C2B3H7B6n_U | |
| Child with leukemia | 4:31 | Negative | Mother discusses her child’s journey with leukemia. | https://www.youtube.com/watch?v=gWVOWVgXIm4 | |
| Dog with lesions | 4:37 | Negative | A veterinarian provides medical treatment for a dog. | https://www.youtube.com/watch?v=tcJwFCbqsQM | |
| Dog eye surgery† | 4:19 | Negative | A veterinarian provides medical treatment for a dog. | https://www.youtube.com/watch?v=4nkx5CUfoNw | |
| Huntington’s disease 1 | 4:54 | Negative | A son discusses the life of his mother who has Huntington’s disease and its impact on his life. | https://www.youtube.com/watch?v=Zp6Am82fZ0o | |
| Huntington’s disease 2† | 5:08 | Negative | Video shows the life of a mother with Huntington’s disease. The video consists of background music and certain parts are narrated by her son. | https://www.youtube.com/watch?v=gyQP93EfKH8 | |
| StoryCorps 1 | 4:58 | Negative | An animated video where a couple share their love story. | https://www.youtube.com/watch?v=WNfvuJr9164 | |
| StoryCorps 2 | 1:57 | Negative | An animated video where a male narrates his love story of his fiancé who passed away during the attack on 9/11. | https://www.youtube.com/watch?v=QgGQAr5hmRI | |
| Beaver | 5:43 | Neutral | Sir David Attenborough narrates a beaver documentary. | https://www.youtube.com/watch?v=iyNA62FrKCE | |
| Dali museum tour | 5:03 | Neutral | A man discusses art works of Salvador Dali. | https://www.youtube.com/watch?v=WSLBtmOAvd4 | |
| Dog competition | 4:12 | Neutral | Judges evaluate owner and dog as they perform various tasks. | https://www.youtube.com/watch?v=_hzs2Ebf-W4 | |
| Ducks | 4:54 | Neutral | Documentary of ducks. | https://vimeo.com/86610300 | |
| Fish | 1:23 | Neutral | Sir David Attenborough narrates a documentary about different fish living in the Red Sea. | https://www.youtube.com/watch?v=z7667jwwX00 | |
| Hannah and her sisters | 1:30 | Neutral | Two women shopping in a store. | https://www.youtube.com/watch?v=fHxeOvCwh6E | |
| Interview | 4:14 | Neutral | Two men sitting across from one another discuss the brain and evolution. | https://www.youtube.com/watch?v=fGKdh-3btkQ | |
| Library tour† | 4:53 | Neutral | A man gives a tour of his office. | https://www.youtube.com/watch?v=Buvksf9-q5U | |
| Turtle | 3:54 | Neutral | Sir David Attenborough narrates a documentary of sea creatures. | https://www.youtube.com/watch?v=z7667jwwX00 | |
| Van Gogh tour† | 5:17 | Neutral | Two men discuss paintings of Vincent van Gogh. | https://www.youtube.com/watch?v=gc-2ArYE9NY | |

*Note*. †Videos selected for the mood-induction protocol.

Appendix C: Correlation table for self-reports in young and older adults combined.

| Variable | *n* | *M* | *SD* | 1 | 2 | 3 | 4 | 5 | 6 | 7 | 8 | 9 | 10 | 11 | 12 | 13 | 14 | 15 | 16 |
| --- | --- | --- | --- | --- | --- | --- | --- | --- | --- | --- | --- | --- | --- | --- | --- | --- | --- | --- | --- |
| 1. Positivity of recall | 258 | -0.03 | 0.22 | – |  |  |  |  |  |  |  |  |  |  |  |  |  |  |  |
| 2. Valence T1 | 250 | 71.60 | 18.41 | .176** | – |  |  |  |  |  |  |  |  |  |  |  |  |  |  |
| 3. Valence T2 | 251 | 55.78 | 27.62 | 0.00 | .284** | – |  |  |  |  |  |  |  |  |  |  |  |  |  |
| 4. Valence T3 | 255 | 56.48 | 19.45 | 0.09 | .373** | .314** | – |  |  |  |  |  |  |  |  |  |  |  |  |
| 5. Arousal T1 | 246 | 48.54 | 21.06 | 0.07 | 0.06 | 0.06 | -0.03 | – |  |  |  |  |  |  |  |  |  |  |  |
| 6. Arousal T2 | 249 | 47.08 | 22.67 | 0.09 | 0.11 | .259** | 0.02 | .320** | – |  |  |  |  |  |  |  |  |  |  |
| 7. Arousal T3 | 248 | 48.47 | 18.97 | .142* | 0.08 | 0.10 | 0.11 | .314** | .412** | – |  |  |  |  |  |  |  |  |  |
| 8. PANAS Positive T1 | 257 | 1.48 | 0.11 | 0.10 | .415** | .139* | .180** | .261** | 0.09 | .227** | – |  |  |  |  |  |  |  |  |
| 9. PANAS positive T3 | 257 | 1.45 | 0.14 | .133* | .337** | .165** | .329** | .186** | .127* | .295** | .831** | – |  |  |  |  |  |  |  |
| 10. PANAS negative T1 | 257 | 1.13 | 0.13 | -0.08 | -.336** | -0.10 | -.249** | .151* | -0.06 | -0.04 | -0.10 | -.146* | – |  |  |  |  |  |  |
| 11. PANAS negative T3 | 257 | 1.13 | 0.14 | -0.04 | -.259** | -.129* | -.409** | 0.09 | 0.02 | 0.05 | -0.09 | -.147* | .713** | – |  |  |  |  |  |
| 12. FTH total | 257 | 46.21 | 12.37 | -.125* | .133* | 0.05 | .151* | -0.03 | -0.05 | -0.02 | .166** | 0.12 | -0.05 | -0.06 | – |  |  |  |  |
| 13. FTH ambiguous | 257 | 14.54 | 5.12 | -0.11 | -.399** | -0.11 | -.247** | -0.02 | -0.09 | -.131* | -.317** | -.323** | .267** | .258** | -.167** | – |  |  |  |
| 14. ERQ appraisal | 257 | 30.39 | 6.20 | 0.02 | .226** | 0.09 | .153* | -0.09 | .144* | .126* | .246** | .273** | -.251** | -.227** | .265** | -0.11 | – |  |  |
| 15. ERQ suppression | 257 | 13.82 | 5.04 | -0.04 | -.279** | -0.10 | -.155* | 0.02 | 0.05 | 0.03 | -.187** | -.175** | .220** | .217** | -0.07 | .249** | -0.09 | – |  |
| 16. CES-D | 256 | 14.52 | 10.43 | -0.10 | -.500** | -0.10 | -.333** | -0.02 | -0.11 | -0.12 | -.335** | -.353** | .572** | .536** | -0.07 | .397** | -.372** | .354** | – |

*Note.* T1 = baseline; T2 = after video; T3 = after memory task. Uncorrected correlation values for the Positive and Negative Affect Schedule (PANAS), Future Time Perspective total score (FTP-total), Future Time Perspective ambiguous subscore (FTP-ambiguous), Emotion Regulation Questionnaire cognitive appraisal component (ERQ-appraisal), emotional suppression component (ERQ-suppression) and Centre for Epidemiologic Studies Depression scale (CES-D). **Correlation is significant at the uncorrected 0.01 level (2-tailed). *Correlation is significant at the uncorrected 0.05 level (2-tailed).
